# Exogenous γ-Aminobutyric Acid (GABA) Enhanced Response to Abiotic Stress in *Hypsizygus marmoreus* by Improving Mycelial Growth and Antioxidant Capacity

**DOI:** 10.3390/metabo14020094

**Published:** 2024-01-27

**Authors:** Zhi Cao, Hongyu Chen, Chenli Zhou, Ming Gong, Yan Li, Youran Shao, Yingying Wu, Dapeng Bao

**Affiliations:** 1 School of Health Science and Engineering, University of Shanghai for Science and Technology, Shanghai 200093, China; 2 National Engineering Research Center of Edible Fungi, Key Laboratory of Applied Mycological Resources and Utilization of Ministry of Agriculture, Institute of Edible Fungi, Shanghai Academy of Agricultural Sciences, Shanghai 201403, China

**Keywords:** *Hypsizygus marmoreus*, GABA, exogenous addition, mycelial growth, antioxidant enzyme

## Abstract

γ-Aminobutyric (GABA) acid is a nutrient and signaling molecule existing in many plants, participating in the regulation of metabolism and various physiological activities. Two strains of *Hypsizygus marmoreus* (a white variety and a brown variety) were investigated to study the impact of exogenous GABA on mycelial growth and the response to stress. Mycelial growth, microscopic morphology, antioxidant profile, and *gad2* expression in *H. marmoreu* were investigated under salt, dehydration, or cold stress. The results indicated that 5 mM GABA stimulated mycelial growth under standard cultivation conditions, whereas GABA addition over 10 mM hindered the growth. Under salt, dehydration, or cold stress, treatment with 5 mM GABA significantly enhanced the mycelial growth rate and density of both *H. marmoreus* strains by promoting front hyphae branching. Meanwhile, the activities of key antioxidant enzymes such as peroxidase (POD), catalase (CAT), and ascorbate peroxidase (APX) were enhanced by GABA, thereby augmenting the defensive network against abiotic stress. Additionally, *gad2* expression and GABA concentration were increased under abiotic stresses as a resistance regulation response. The exogenous addition of GABA strengthened the upregulation of *gad2* expression and GABA production. These findings indicated that exogenously adding low concentrations of GABA effectively enhanced the mycelial growth and antioxidant profile of *H. marmoreus*, thereby improving its resistance against stresses.

## 1. Introduction

γ-Aminobutyric acid (GABA, molecular formula C_4_H_9_O_2_N) is a non-protein amino acid which widely found in plants, animals, bacteria, and fungi. In 1949, Stewart et al. [1] first reported the presence of GABA in potato tuber tissue. Subsequently, Roberts et al. [2] and Awapara et al. [3] independently discovered GABA in the brains of mice and rats, respectively. After nearly 50 years of research, the in vivo functions of GABA have been gradually clarified. In animals, GABA serves as an inhibitory neurotransmitter and a versatile signaling molecular in the central nervous system, peripheral nervous system, and some non-neuronal tissues. GABA is hailed as a natural tranquilizer with the ability to improve symptoms of anxiety and depression. Its working mechanism consists in binding to the GABA_A_ receptor, thereby opening specific chloride ion channels and inducing neuronal hyperpolarization and shunting inhibition. This process prevents signals associated with anxiety from reaching the central command center of the brain [4]. GABA regulates blood pressure by promoting vasodilation and inhibiting the activities of angiotensin-converting enzymes, thereby reducing blood pressure levels [5,6]. It also exhibits immune-modulating functions [7] and anti-cancer effects such as the suppression of the proliferation and metastasis of breast [8] and colon cancer cells [9,10]. GABA is closely associated with the regulation of hypothalamic functions, contributing to memory improvement, hormonal regulation [11], and the alleviation of neuroexcitatory disorders such as epilepsy [12]. Previous studies also confirmed the therapeutic effects of GABA in treating asthma [13] and pancreatic dysfunction [14]. In higher plants, GABA is present in various organs throughout the developmental stages [15]. This distinctive abundance stands in stark contrast to the highly restricted occurrence of other non-protein amino acids, underscoring its uniqueness. *Arabidopsis thaliana* exhibited robust growth when cultivated on a medium where GABA served as the sole nitrogen source, indicating that GABA provides nutritional support to plants [16]. Moreover, extensive research confirmed that GABA functions as a signaling molecule in regulating plant growth and development, promoting seed germination [17], and enhancing seedling growth [18]. GABA also modulated the intracellular pH in plant cells, contributing to cytoplasmic acidification [18]. Additionally, GABA plays a positive role in symbiotic regulation [19] and in the regulation of the synthesis and metabolism of plant hormones such as abscisic acid [20].

In recent years, the role of GABA in mitigating abiotic stress in plants and microorganisms has attracted increased research interest. Previous studies showed that the content of GABA in the organism changes rapidly under abiotic stress [21,22,23], suggesting that GABA may play a special role in regulating plant tolerance to abiotic stresses. Yang et al. [24] reported that GABA synthesis under hypoxic stress consumes protons, thereby regulating the intracellular pH. The synthesized GABA enters the TCA cycle through the GABA shunt to maintain the carbon–nitrogen balance in plants, reducing the damage caused by hypoxic stress. Ji et al. [25] discovered that GABA accumulation played a crucial role in enhancing *Candida glycerinogenes* resistance to high osmolarity. A growing number of studies have shown that the exogenous application of low concentrations of GABA increased the levels of endogenous GABA and elicited a variety of biochemical, molecular, and physiological cascades, thereby increasing the tolerance of plants and microorganisms to diverse abiotic stressors [26,27,28]. Chen [29] found that GABA alleviated salt stress during seed germination by increasing Na^+^/K^+^ transport, promoting dehydration, and regulating the osmotic pressure. Exogenous GABA promotes the accumulation of abscisic acid (ABA) in plants, activates the ABA signaling pathway, induces stomatal closure, and enhances the drought stress tolerance of apples [30]. In addition, exogenously added GABA is able to activate the TCA cycle to produce more energy and metabolic intermediates such as succinic acid. It also acts as an enzyme activator, enhancing the activity of antioxidant enzymes and of enzymes scavenging reactive oxygen species, thereby reducing oxidative damage and alleviating the adverse effects of stress. The exogenous addition of GABA increased the activities of many antioxidant enzymes, such as Superoxide dismutase (SOD), CAT, and POD, in tomato [31], rice [32], and *Medicago sativa* L. [33], which effectively protected the above plants against the oxidative damage induced by salt, alkali, and high temperature. These studies suggest that the exogenous addition of low concentrations of GABA would be a potential method to improve the resistance of plants and edible mushrooms.

There are two main pathways for GABA production, one of which is the GABA synthesis branch in the TCA cycle. In this pathway, alpha-ketoglutarate (α-KG) in the TCA cycle is catalyzed by glutamate dehydrogenase (GDH) to produce L-Glu; subsequently, an irreversible decarboxylation reaction at the α-site of L-Glu is catalyzed by GAD to produce GABA [34]. The second pathway is the polyamine degradation in plants, where putrescine, spermine, and spermidine generate 4-aminobutyric acid and 4-(3-aminopropyl)-aminobutyraldehyde by the action of polyamine oxidases and diamine oxidases, which are then dehydrated and converted to GABA by pyrrolidinium dehydrogenase [35]. Glutamate acid decarboxylase (GAD) is a 5′-phosphatepyridoxal (PLP)-dependent enzyme widely found in plants, animals, and microorganisms. GAD specifically catalyzes the decarboxylation of L-glutamate to GABA and is considered a key enzyme in GABA synthesis [34]. GAD genes have been cloned and identified in many plants such as *Camellia sinensis* [36], citrus [37], and tomato [38], where GAD appeared as a regulator of growth and development. Studies showed that GAD is the most sensitive gene in the process of GABA response to abiotic stress, and the level of GAD expression is closely related to GABA-mediated enhancement of plant stress tolerance [39]. Moreover, the GABA levels are regulated by GAD transcript expression. A significant increase in GABA accumulation and GAD mRNA levels under salt and osmotic stress was observed in five wheat cultivars [40]. Hu et al. [41] reported that the *CsGAD* gene enhanced salinity tolerance in melon by increasing leaf GAD activity and GABA content. However, most of the studies on GABA in microorganisms and fungi focused on increasing GABA production through fermentation [42], and few studies examined the relationship between GAD, GABA, and stress tolerance in macrofungi, especially, edible fungi.

*H. marmoreus* is a lignicolous saprophytic macrofungus taxonomically classified into the *Basidiomycotina* phylum, the *Agaricomycetes* class, *the Agaricales* order, and the *Lyophyllaceae* family. *H. marmoreus* is rich in nutrients such as various vitamins, minerals, proteins, and amino acids [43]. Recent studies confirmed that it contains small molecular compounds, polysaccharides, and short peptides with various health benefits, including antioxidative properties [44], anti-tumor proliferation [45], antibacterial, and anti-inflammatory activities [46], and blood sugar regulation [47] and immunomodulation [48] properties. According to statistics from the China Edible Fungi Association, the total production of *H. marmoreus* reached 526,300 tons, ranking fourth among those of cultivated edible fungi in China and showing that *H. marmoreus* has tremendous market potential. The yield and quality of *H. marmoreus* are highly influenced by various abiotic stress factors during the cultivation process, such as temperature, osmotic pressure, and salinity. Enhancing the resistance of *H. marmoreus* strains to abiotic stress is an effective approach to improve *H. marmoreus* yield. This study explored the physiological regulation induced by GABA in *H. marmoreus* strains in response to abiotic stress based on the analysis of mycelial growth, microscopic morphology, antioxidant profile, and *gad2* expression. The findings of this study propose a novel direction for enhancing strain resistance, as well as provide reference for breeding and producing high-quality edible fungi with improved stress tolerance.

## 2. Materials and Methods

### 2.1. Materials

#### 2.1.1. Strains

The *H. marmoreus* strains (*NN12*, *W3*) used in this study are preserved in the Institute of Edible Fungi, Shanghai Academy of Agricultural Sciences.

#### 2.1.2. Culture Medium

Potato dextrose agar (PDA) medium: 4 g of potato starch, 20 g of glucose, and 15 g of agar were dissolved into distilled water to prepare 1 L of medium solution. After autoclaving at 121 °C for 20 min, 20 mL of the medium was poured into a 90 mm Petri dish.

GABA at different concentrations (5, 10, 15, and 20 mM) was added into the PDA medium to test the effects of GABA on mycelial growth under various conditions. NaCl at different concentrations (50, 100, 150, 200, 300, and 400 mM) was added to induce salt stress. C_6_H_14_O_6_ at different concentrations (100, 200, 300, 400, and 500 mM) was added to induce dehydration stress.

### 2.2. Methods

#### 2.2.1. Determination of Mycelial Growth Rate

The mycelial growth rate was measured using the cross-marking method [49]. After inoculation, a cross was drawn on the back of each Petri dish to mark the center. The dishes were cultured in a 23 °C incubator. As the mycelium began to grow and elongate, the living edge of the mycelium was marked. After 6 days of culture, another mark was placed on the updated living edge. The average mycelial growth rate was calculated by dividing the distance between the two marks by the number of days of culture.

#### 2.2.2. Quality Inspection of the Fresh Aerial Mycelia

Following the method described by You [50], a cellulose acetate membrane was spread on the culture medium, ensuring its close adhesion without hindering mycelial growth. A culture block was inoculated in the membrane center. After incubating in different conditions for 14 days, the cellulose acetate membrane was removed, and the aerial mycelium was collected. An electronic analytical balance (Shanghai, China) was used to precisely determine the fresh weight of the aerial mycelium.

#### 2.2.3. Microscopic Observation of the Mycelia

Mycelium blocks were inoculated in the center of the culture medium, and a sterilized disposable cover glass was inserted at a 45° angle, 1 cm away from each block. The dishes were cultured in different conditions until the living edges of the mycelium had climbed up 2/3 of the cover glass. Then, the slides were observed using an inverted microscope (Guangzhou, China). Fifty fields in each microscope view were randomly selected, the diameter and number of septate junctions in each field were measured.

#### 2.2.4. Determination of the GABA Content

The cultured mycelium was collected and transferred into a mortar, and then grinded in powder liquid nitrogen. The mycelium powder was transferred into a 2 mL centrifuge tube, and the GABA content was measured using a rapid assay kit purchased from Enzyme-Linked Biotechnology.

#### 2.2.5. Determination of Antioxidant Enzyme Activity

SOD activity was determined referring to the nitroblue tetrazolium method [51]. One unit of SOD activity is defined as the amount of enzyme required for a 50% inhibition rate in the reaction system. The activity of APX was measured by ultraviolet spectrophotometry [52]; one unit is defined as the enzyme amount catalyzing the oxidation of 1 μmol ascorbic acid per gram of sample per minute. The reagents were obtained from Beijing Box Biological Science and Technology Co., Ltd. (Beijing, China).

POD activity was determined referring to the guaiacol method [52]; one unit is defined as the enzyme amount required to catalyze the oxidation of 1 gram of sample in each mL of the reaction system per minute. CAT activity was detected by UV spectrophotometry [53]; one unit is defined as the enzyme amount catalyzing the degradation of 1 μmol H_2_O_2_ per gram of sample per minute in the reaction system. The relevant reagents were purchased from Shengong Bioengineering (Shanghai, China) Co., Ltd.

#### 2.2.6. Determination of *gad2* Expression

After the mycelial samples of *H. marmoreus* were ground in liquid nitrogen, total RNA was extracted using the RNA Isolater Extraction kit. The RNA concentration and purity were determined using a Nanodrop spectrophotometer, with the A260/280 ratio required to be in the range of 1.8–2.0. The RNA was reverse-transcribed into cDNA using HiScript II Q RT SuperMix for qPCR, and qRT-PCR analysis was conducted using ChamQ Universal SYBR qPCR Master Mix. All relevant reagents were purchased from Novogene Biotech Co., Ltd., Nanjing, China. Specific primers were designed for *H. marmoreus HM62* strain GAD gene (GenBank: CM024084.1) based on the NCBI database, with the *H. marmoreus* actin gene selected as the reference gene (Table 1). The 2^−ΔΔCT^ method [54] was employed to calculate the relative gene expression levels. The expression level at 23 °C of the control group was recorded as 1.

### 2.3. Data Analysis

Data processing and analysis were conducted using Excel and IBM SPSS Statistics 25 software. All experiments were performed in triplicate. Significant differences were analyzed using one-way ANOVA and are presented as significant (*p* < 0.05) and highly significant (*p* < 0.01). Visual processing was performed using Photoshop 2020 and GraphPad 8.0 software.

## 3. Results and Analysis

### 3.1. Effect of Exogenous GABA or Abiotic Stress Factors on Mycelial Growth under Standard Culture Conditions

**Figure 1:**
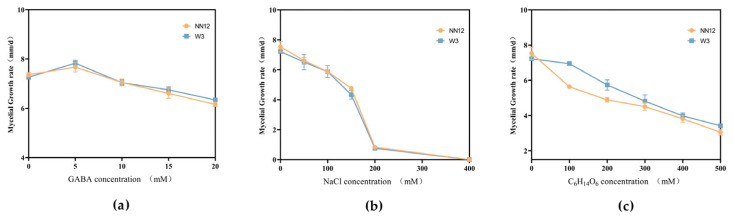
Mycelial growth rates of the strains *NN12* and *W3*. (**a**) Addition of different concentrations of GABA; (**b**) addition of different concentrations of NaCl; (**c**) addition of different concentrations of C_6_H_14_O_6_.

To explore the impact of exogenously added GABA on the mycelial growth of *H. marmoreus*, four concentrations of GABA (5, 10, 15, 20 mM) were separately added into standard PDA medium. The colony morphology of the strains (*NN12* and *W3*) was observed, and the mycelial growth rate was recorded. The results indicated that adding a low concentration (5 mM) of GABA promoted mycelial growth, leading to larger colony diameters, and the growth rates of *NN12* and *W3* were increased by 3.65% ± 0.51% and 6.59% ± 0.99%, respectively. Adding higher concentrations (10, 15, 20 mM) of GABA inhibited mycelial growth, and the inhibitory effect became more pronounced as the concentration increased. Compared to the blank control, the inhibition of the mycelial growth of *NN12* and *W3* was 13.11% ± 1.17% and 11.95% ± 0.71%, respectively, after treatment with 20 mM GABA (Figure 1a and Figure S1I). Therefore, 5 mM was selected as the optimized concentration for the subsequent experiments.

Simultaneously, six concentrations of sodium chloride (NaCl), i.e., 50, 100, 150, 200, 300, and 400 mM, or five concentrations of mannitol (C_6_H_14_O_6_), i.e., 100, 200, 300, 400, and 500 mM, were separately added to the PDA solid medium to observe their effects on the growth of the *NN12* and *W3* mycelia. The results demonstrated that different concentrations of NaCl and C_6_H_14_O_6_ inhibited mycelial growth, with a typical dose–response relationship, where higher concentrations led to a stronger inhibition. When NaCl concentration reached 400 mM, mycelial growth was completely suppressed (Figure 1b). When *NN12* and *W3* were treated with 500 mM of C_6_H_14_O_6_, the inhibition rates reached 59.47% ± 0.26% and 52.64% ± 0.94%, respectively (Figure 1c). Based on colony growth morphology (Figure S1II,III) and mycelial growth rate, 150 mM NaCl and 300 mM C_6_H_14_O_6_ were determined as the concentrations for the subsequent experiments to induce salt stress and dehydration stress.

### 3.2. Effect of Exogenous GABA on Mycelial Growth under Stress

**Figure 2:**
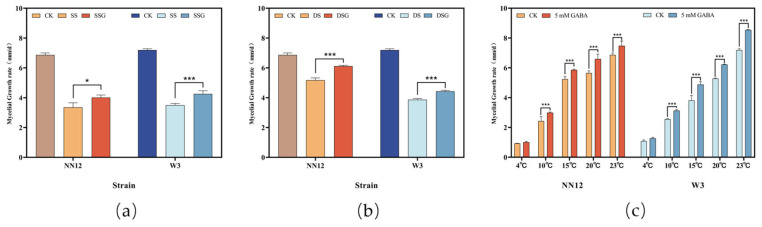
Effect of 5 mM GABA on the mycelial growth rates under abiotic stress. (**a**) Mycelial growth rates of *NN12* and *W3* under salt stress; (**b**) mycelial growth rates of *NN12* and *W3* under dehydration stress; (**c**) mycelial growth rates of *NN12* and *W3* at various temperatures. Bars represent mean ± SE (*n* = 3). Regarding the statistical differences, *p*-values < 0.05 are designated with one (*) asterisk, and *p*-values < 0.01 are designated with three (***) asterisks. CK: control; SS: 150 mM NaCl; SSG: 150 mM NaCl + 5 mM GABA; DS: 300 mM C_6_H_14_O_6_; DSG: 300 mM C_6_H_14_O_6_ + 5 mM GABA.

**Figure 3:**
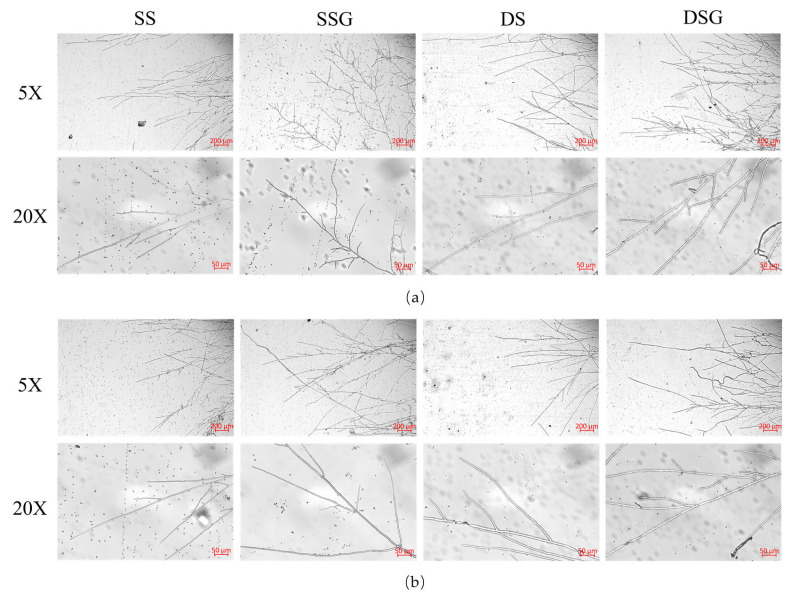
Effect of GABA on the microscopic morphology of the mycelia under abiotic stress. (**a**) Strain *NN12*; (**b**) strain *W3*. SS: 150 mM NaCl; SSG: 150 mM NaCl + 5 mM GABA; DS: 300 mM C_6_H_14_O_6_; DSG: 300 mM C_6_H_14_O_6_ + 5 mM GABA.

To explore the impact of exogenous GABA addition on the mycelial growth of *H. marmoreus* under salt and dehydration stresses, 5 mM GABA + 150 mM NaCl or 5 mM GABA + 300 mM C_6_H_14_O_6_ were added into the PDA medium. The colony and microscopic morphologies were observed, and the growth rate and fresh weight of the mycelium were recorded. The results indicated that exogenously adding 5 mM GABA significantly increased the mycelial growth rate (*p* < 0.05) (Figure 2a) and fresh weight (*p* < 0.01) (Figure S3(1)) of *NN12* and *W3* under salt and dehydration stresses. The rate of *NN12* mycelial growth increased by 20.21% ± 5.75% under salt stress and by 18.44% ± 2.53% under dehydration stress, while the mycelial growth rate for W3 increased by 21.73% ± 2.29% and 14.69% ± 1.13%, respectively. Additionally, the mycelium treated with 5 mM GABA grew denser, and the colonies were larger (Figure S2). Microscopic observation of both strains (Figure 3) revealed an increase in the number of secondary branches and clamp connections after adding 5 mM GABA, accompanied by a slight reduction in the mycelium tip diameter (Table S1).

To explore the impact of exogenously adding GABA on the mycelial growth of *H. marmoreus* in low-temperature environments, 5 mM GABA was added to the PDA medium. After inoculation, the cultures were incubated at 4, 10, 15, and 20 °C and compared to cultures at the optimal temperature of 23 °C. The colony and microscopic mycelial morphologies were observed, and the mycelial growth rates were recorded. The results indicated that the mycelial growth rate was the highest at 23 °C and slowed down as the temperature decreased. The exogenous addition of 5 mM GABA significantly increased (*p* < 0.01) the mycelial growth rate of both strains at 10, 15, 20, and 23 °C (Figure 2b,c). The most significant enhancements for *NN12* and *W3* were observed at 10 °C and 15 °C, respectively. The mycelial growth rate of *NN12* at 10 °C was improved by 23.74% ± 13.75%, and that of W3 at 15 °C was elevated by 27.94% ± 6.20%. Additionally, at higher temperatures, the mycelium grew denser, forming larger colonies (Figure S2) with higher fresh weight (Figure S3(2,3)).

### 3.3. Effect of Exogenous GABA on Mycelial Antioxidant Capacity under Stress

**Figure 4:**
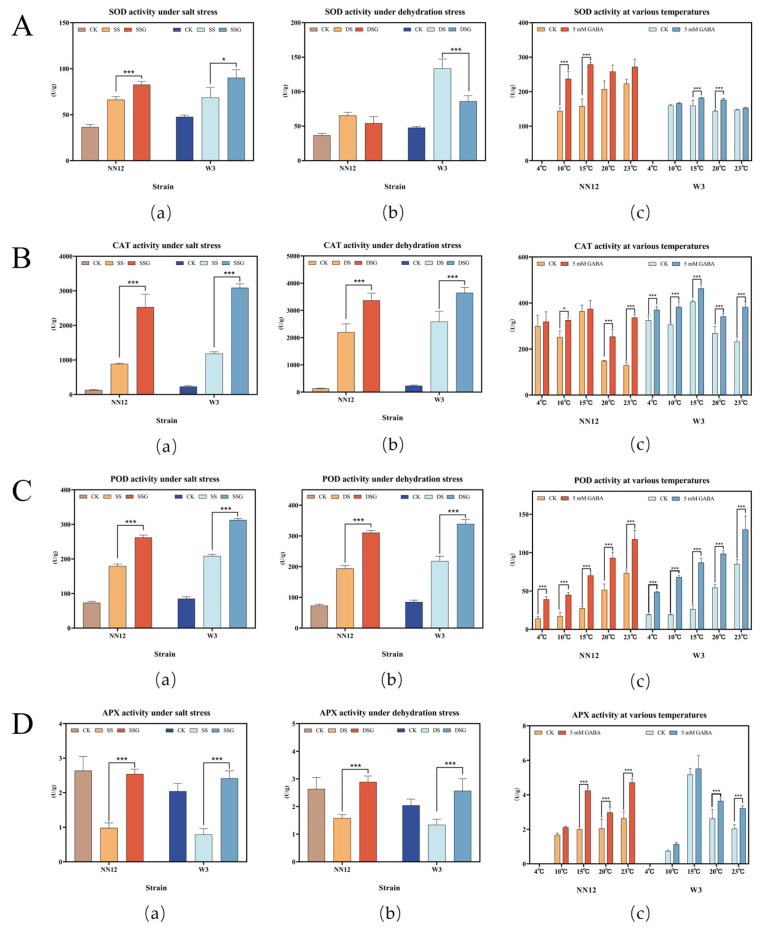
The mycelial antioxidant enzyme activity after adding 5 mM GABA under abiotic stress. (**A**) SOD activity; (**B**) CAT activity; (**C**) POD activity; (**D**) APX activity. (**a**) Enzyme activity of mycelium under salt stress; (**b**) enzyme activity of mycelium under dehydration stress; (**c**) enzyme activity of mycelium under low-temperature stress; The bars represent the mean SE (*n* = 3). Regarding the statistical differences, *p*-values < 0.05 are designated with one (*) asterisk, and *p*-values < 0.01 are designated with three (***) asterisks. CK: control; SS: 150 mM NaCl; SSG: 150 mM NaCl + 5 mM GABA; DS: 300 mM C_6_H_14_O_6_; DSG: 300 mM C_6_H_14_O_6_ + 5 mM GABA.

The results indicated that the activities of three enzymes (SOD, CAT, and POD) were upregulated under salt or dehydration stresses (Figure 4A–C), while APX activity was reduced (Figure 4D). The exogenous addition of 5 mM GABA effectively stimulated the activity of the above antioxidant enzymes, further strengthening the defensive network against abiotic stress (*p* < 0.01). The exogenous addition of 5 mM GABA significantly increased POD activity in both strains adapting to temperature variation (*p* < 0.01) (Figure 4C). At 15 °C, SOD activity was improved in both strains after GABA supplementation (Figure 4A). CAT activity was significantly increased in *W3* at all tested temperatures and in *NN12* at 20 °C and 23 °C (Figure 4B). APX activity was increased in both strains at 20 °C and 23 °C (Figure 4D). These findings reflect the effectiveness of GABA in enhancing the strains’ antioxidant capacity, with variance existing in the sensitivity to stress in different strains and for different enzymes.

One mechanism related to antioxidant resistance involves ROS accumulating significantly within the *H. marmoreus* mycelium under stress conditions, concurrently activating the antioxidant enzyme system. This activation leads to increased activities of SOD, CAT, and POD, along with decreased APX activity. The exogenous addition of 5 mM GABA facilitated the activation of the mycelial antioxidant enzyme system. As compared with *NN12*, the strain *W3* was more responsive to stress and GABA.

### 3.4. Effects of Exogenous GABA on Endogenous GABA Content and gad2 Expression Levels in Mycelia under Stress

**Figure 5:**
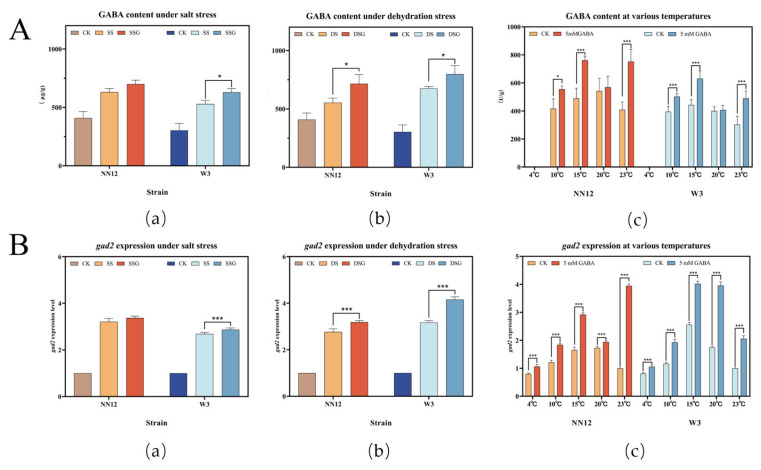
Effect of 5 mM GABA on mycelial GABA content (**A**) and *gad2* gene expression levels (**B**) under abiotic stress. (**a**) GABA content (**A**) or *gad2* expression(**B**) of mycelium under salt stress; (**b**) GABA content (**A**) or *gad2* expression (**B**) of mycelium under dehydration stress; (**c**) GABA content (**A**) or *gad2* expression (**B**) of mycelium under low-temperature stress; The bars represent the mean SE (*n* = 3). Regarding the statistical differences, *p*-values < 0.05 are d esignated with one (*) asterisk, and *p*-values < 0.01 are designated with three (***) asterisks. CK: control; SS: 150 mM NaCl; SSG: 150 mM NaCl + 5 mM GABA; DS: 300 mM C_6_H_14_O_6_; DSG: 300 mM C_6_H_14_O_6_ + 5 mM GABA.

To investigate GABA content and *gad2* expression in *H. marmoreus*, the mycelium was scraped for GABA concentration analysis and real-time fluorescence quantitative PCR analysis. The results indicated that under salt and dehydration stresses, the endogenous GABA content in the mycelia of both *NN12* and *W3* strains significantly increased (*p* < 0.05), indicating GABA was an active regulator in response to abiotic stress. The exogenous addition of 5 mM GABA further induced an increase in the endogenous GABA content of both strains under salt and dehydration stress (Figure 5A(a,b)). Specifically, under salt stress, the endogenous GABA content in *NN12* increased by 14.62% ± 3.72%, and that in *W3* by 19.18 ± 1.33% as compared to the control. Under dehydration stress, *NN12* showed an increase of 29.20% ± 4.80% in GABA content, and *W3* showed an increase of 18.24% ± 9.04%. Additionally, the exogenous addition of 5 mM GABA significantly increased the endogenous GABA content in the *NN12* and *W3* strains under cold stress at 10 °C and 15 °C, as well as under the optimal temperature condition of 23 °C (*p* < 0.05).

Under salt stress and dehydration stress, the expression levels of *gad2* in the mycelia of *NN12* and *W3* significantly increased (*p* < 0.01). The exogenous addition of 5 mM GABA further significantly elevated *gad2* expression in *W3* under salt stress and in both strains under dehydration stress (*p* < 0.01) (Figure 5B(a)). The expression of *gad2* in the mycelia varied at different temperatures. The exogenous addition of 5 mM GABA significantly increased *gad2* expression in both strains at various temperatures (*p* < 0.05). The most significant increase was observed in *NN12* at 23 °C (292.67% ± 8.08%), while *W3* showed the most significant increase at 20 °C (126.00% ± 3.11%).

The results indicated that the stressful environments led to the upregulation of *gad2* expression and the accumulation of endogenous GABA within the mycelia. The exogenous addition of GABA further increased *gad2* expression. These results suggest a strong correlation between *gad2* expression and GABA generation.

## 4. Discussion

During the growth process, plants inevitably encounter different types of stress, caused by both biotic and abiotic factors. Abiotic stress refers to adverse effects caused by various non-living factors in specific environments, leading to changes in biological processes such as growth and development. Abiotic stress types primarily encompass high temperature, low temperature, drought, salt, and metal stress [55]. Global climate changes increase the complexity of crop growth, posing abiotic stressor-related threats and constraints to modern agriculture. Researchers globally are actively pursuing convenient and effective methods to augment crop resilience, consequently elevating the agricultural productivity [56]. Edible fungi have become pivotal agricultural commodities, and the mushroom industry displays promising market potential and extensive expansion prospects [57]. During the cultivation cycle of edible fungi, factors such as temperature, humidity, substrate salinity, and osmotic pressure significantly impact yield and quality [58]. Enhancing the stress resistance of mushroom strains stands as an effective strategy to promote the efficient development of the mushroom industry.

Edible fungi are heterotrophic eukaryotes lacking chlorophyll, which utilize extracellular enzymes secreted by the growth tips of the mycelium to decompose nutrients in the substrate for growth and development. Similar to other cells, the fungal mycelium exhibits high sensitivity to the environment [59]. Under favorable conditions, mycelial life activities proceed as normal; yet in stress environments, mycelial vigor is restrained, leading to slowed growth. Zhang et al. [60] reported that when solid medium was treated with stress factors such as CdCl_2_ (50 µM), NaCl (1.0%), and H_2_O_2_ (2 mM), the mycelial growth of *H. marmoreus* was reduced, and the mycelial volume decreased by about 50%. Our study revealed a significant reduction in the growth rate of *H. marmoreus* mycelium under low-temperature, dehydration, and salt stress. Moreover, the more severe the stress environment, the more pronounced the inhibition on mycelial growth. Among the types of stress, both salt treatment (NaCl addition) and dehydration treatment (C_6_H_14_O_6_ addition) induced changes in the osmotic pressure of the medium. According to van’t Hoff’s law, the osmotic pressure of a solution is directly proportional to the concentration of the dissolved particles but is not dependent on the solute nature. The estimated osmotic pressure of the dehydration stress medium (680.9166 Kpa) was twice that of the salt stress medium (340.4583 Kpa) at 25 °C. However, we observed that the inhibitory effect of salt stress on mycelial growth was more pronounced than that of dehydration stress in this experiment, which might have been caused by the stronger toxic effect of Cl^-^ from NaCl.

Several studies found that the addition of low concentrations of GABA improved or promoted the growth of plants. Kinnersley et al. [61] reported that the addition of 5 mM GABA was able to increase the growth of *Lemna minor* L. by 2–3 times. Wu et al. [31] reported that the exogenous addition of 5 Mm GABA had a positive effect on tomato seedlings against salt stress, improving plant height, chlorophyll content, and dry fresh weight. Different from plants, the growth indicators of edible fungal mycelium are primarily reflected in metrics such as growth rate and the number of clamp connections. The more frequent clamp connections are observed, the more active cell division and hyphal extension are, indicating better growth potential and stronger resistance. This study found that the exogenous addition to *H. marmoreus* of 5 mM GABA effectively enhanced the growth rate and aerial weight of mycelium in three stressful environments. The microscopic morphology of the mycelium under salt and dehydration stress was also altered, with a reduction in the diameter of the mycelium tips and an increase in the number of branches at the mycelium tips and in the number of clamp connections. Similar to the results obtained when treating plants with the same concentration of GABA, the findings of this study demonstrated that the exogenous addition of 5 mM GABA could effectively promote the growth of *H. marmoreus* mycelium. Hijaz et al. [62] speculated that the mechanism by which GABA promotes plant growth may be related to its temporary role as a nitrogen source under stress conditions and to the activation of the GABA shunt and TCA cycle to produce more energy, thereby improving the growth condition of plants under stress. Hitherto, the mechanism by which exogenous GABA promotes the growth of edible fungal mycelium is not fully clear and is worth further in-depth study.

The antioxidant enzyme system is a defense system evolved in higher plants against external stresses [63] and is also present in edible mushrooms. Reactive oxygen species (ROS) are byproducts of cellular metabolism. In normal growth conditions, ROS production and scavenging maintain a dynamic equilibrium [64]. When edible fungi are exposed to stress, this balance is disrupted, triggering the activation of defense systems such as antioxidant enzymes to neutralize excessive ROS and restore the equilibrium. However, when the fungi own defense systems are insufficient to counter stress, ROS accumulation occurs, severely affecting mycelial growth and potentially leading to cell death [65]. SOD, CAT, and POD are the primary antioxidant enzymes in edible fungi. SOD is the first line of enzymatic defense against ROS, catalyzing the dismutation of O^2-^ to generate O_2_ and H_2_O_2_. It plays a crucial role in the reactive oxygen species scavenging system and is also an important indicator of stress resistance [66]. The function of CAT and POD is to eliminate the vast majority of H_2_O_2_, preventing cellular oxidative injury. Li et al. [33] reported that spraying 75 mM GABA increased antioxidant enzyme activities (SOD, CAT, POD) in *Medicago sativa* L. under alkaline stress. Similar to the findings in plants, this study discovered that under various stresses, the activities of SOD, CAT, and POD in *H. marmoreus* mycelium sharply increased. It may be speculated that, as in the case of oxidative damage caused by a stressful environment, the mycelium activates a defense response against potential damage by increasing the activity of antioxidant enzymes. Under salt stress and dehydration stress conditions, the activity of APX in the mycelium of two strains decreased. This could be because APX is not only an antioxidant enzyme but also one of the key enzymes in ascorbic acid metabolism [67]. Under stress conditions, the mycelial metabolism was weakened, thus reducing APX activity. Additionally, the changes in enzyme activity under salt stress and dehydration stress were greater than under low temperature, indicating that low temperature had a relatively smaller impact on the antioxidant enzyme system in *H. marmoreus* mycelium. Combined with the results of mycelial growth improvement, it is suggested that GABA is an effective signaling molecule that allows *H. marmoreus* to adapt to environmental stresses; its mechanism of action was also associated in vivo with the antioxidant defensive system.

Both abiotic stress and exogenous addition affect the endogenous in vivo GABA levels, and intrinsic mechanisms are related to the metabolic pathway of GABA. The synthetic pathways of GABA include the GABA shunt and the polyamine degradation pathways. Of the GABA accumulated under adverse conditions, 61% originates from the GABA shunt, and 39% from the polyamine degradation pathway [68]. Numerous studies confirmed that the expression level of the gene encoding the key enzyme GAD in the GABA shunt is related to the GABA levels. Bao et al. [69] reported that after exposure to 200 mM NaCl for 5 days, the GABA levels in the leaves of tomato plants increased by twofold. Li et al. [70] reported that the exogenous addition of 0.5 mM GABA raised the endogenous GABA content in the apomictic *Malus hupehensis* leaves under normal and alkaline stress, while *GAD1* and *GAD2* expression was significantly upregulated under alkaline stress. Cheng et al.’s research [71] indicated that the exogenous addition of 1 mM GABA upregulated *MdGAD1*, *MdGAD2*, *MdGAD3*, and *MdGAD4* expression in the dwarf apple rootstock *M.9-T337* under drought stress. Aydin et al. [72] found that the exogenous addition of 0.1 mM GABA upregulated *GAD* expression in white button mushrooms, consequently increasing the endogenous GABA content. This study not only confirmed the significant increase in endogenous GABA content in *H. marmoreus* mycelium under three types of environmental stresses but also found that the exogenous addition of 5 mM GABA further enhanced the endogenous GABA accumulation. Under salt stress and dehydration stress, 5 mM exogenous GABA increased the mycelial GABA content by more than 1.5 times with respect to that in the negative control, indicating a close relationship between GABA metabolism and the mycelium response to adverse conditions. In our preliminary bioinformatics analysis, we identified the gene *gad2* in the genome of the monokaryotic strain of *H. marmoreus*, which may be responsible for synthesizing the key enzyme GAD in the GABA synthesis pathway. This study found that after the exogenous addition of 5 mM GABA, the expression level of *gad2* in *H. marmoreus* mycelium significantly increased under three stress conditions, suggesting that the exogenously added GABA could activate the GABA shunt, inducing an increase in endogenous GABA content by upregulating the expression of the key gene *gad2* in the GABA synthesis pathway.

## 5. Conclusions

In summary, this study investigated how exogenous low-concentration GABA affected the resistance of *H. marmoreus* mycelium to abiotic stress. The findings showed that adding 5 mM GABA markedly enhanced the mycelial resistance to salt, dehydration, and low-temperature stresses. This improvement was evident in enhanced mycelial growth rate, increased fresh weight, heightened antioxidant enzyme activities, elevated internal GABA content, and upregulated *gad2* expression. These positive effects might be due to the fact that the exogenous addition of GABA activated the TCA cycle to generate more energy, improved the antioxidant defensive system, reducing oxidative stress damage associated with the ROS levels, or regulated the plant defense-related transporter proteins and channels. These mechanisms collectively strengthened *H. marmoreus* mycelia’s resistance to abiotic stress; yet, further investigations are needed on the specific signaling transduction pathways. These results offer a promising approach to enhance stress tolerance in edible fungi, potentially improving their stress resistance capacities. Moreover, this research lays foundation for future applications, such as utilizing edible fungi as cell factories for GABA production.

## Figures and Tables

**Table 1:** Primer sequences used in real-time qPCR.

Primer	Sequence (5′-3′)
*gad2*-q-Forward	GGTCTCACCAGCACGAAT
*gad2*-q-Reverse	TTGGAAGAGTGTTGTAGCG
ACT-Forward	CCGAGCGGAAGTACTCTGTG
ACT-Reverse	ATGCTATCTTGCCTCCAGCC

## Data Availability

The data presented in this study are available in this article.

## Supplementary Materials

The following supporting information can be downloaded at https://www.mdpi.com/article/10.3390/metabo14020094/s1. Figure S1: Effect of GABA on colony morphology in *H. marmoreus*; Figure S2: Effect of GABA on colony morphology under abiotic stress; Figure S3: Effect of GABA on the fresh weight of aerial mycelium under abiotic stress; Table S1: Mycelial tip diameter under salt stress and dehydration stress.

## References

[1] Steward F.C., Thompson J.F., Dent C.E. (1949). γ-Aminobutyric acid: A constituent of the potato tuber?. Science.

[2] Roberts E., Frankel S. (1950). gamma-Aminobutyric acid in brain: Its formation from glutamic acid. J. Biol. Chem..

[3] Awapara J., Landua A.J., Fuerst R., Seale B. (1950). Free gamma-aminobutyric acid in brain. J. Biol. Chem..

[4] Tafet G.E., Nemeroff C.B. (2020). Pharmacological Treatment of Anxiety Disorders: The Role of the HPA Axis. Front. Psychiatry.

[5] Hayakawa K., Kimura M., Kamata K. (2002). Mechanism underlying γ-aminobutyric acid-induced antihypertensive effect in spontaneously hypertensive rats. Eur. J. Pharmacol..

[6] Yang N.C., Jhou K.Y., Tseng C.Y. (2012). Antihypertensive effect of mulberry leaf aqueous extract containing γ-aminobutyric acid in spontaneously hypertensive rats. Food Chem..

[7] Huang D., Wang Y., Thompson J.W., Yin T., Alexander P.B., Qin D., Mudgal P., Wu H., Liang Y., Tan L. (2022). Cancer-cell-derived GABA promotes β-catenin-mediated tumour growth and immunosuppression. Nat. Cell Biol..

[8] Opolski A., Mazurkiewicz M., Wietrzyk J., Kleinrok Z., Radzikowski C. (2000). The role of GABA-ergic system in human mammary gland pathology and in growth of transplantable murine mammary cancer. J. Exp. Clin. Cancer Res..

[9] Song L., Du A., Xiong Y., Jiang J., Zhang Y., Tian Z., Yan H. (2016). γ-Aminobutyric acid inhibits the proliferation and increases oxaliplatin sensitivity in human colon cancer cells. Tumor Biol..

[10] Fava G., Marucci L., Glaser S., Francis H., De Morrow S., Benedetti A., Alvaro D., Venter J., Meininger C., Patel T. (2005). γ-Aminobutyric acid inhibits cholangiocarcinoma growth by cyclic AMP-dependent regulation of the protein kinase A/extracellular signal-regulated kinase 1/2 pathway. Cancer Res..

[11] Xu Y., Zhao M., Han Y., Zhang H. (2020). GABAergic Inhibitory Interneuron Deficits in Alzheimer’s Disease: Implications for Treatment. Front. Neurosci..

[12] van van Hugte E.J.H., Schubert D., Kasri N.N. (2023). Excitatory/inhibitory balance in epilepsies and neurodevelopmental disorders: Depolarizing γ-aminobutyric acid as a common mechanism. Epilepsia.

[13] Arnold L.A., Forkuo G.S., Nieman A.N., Yu O.B., Guthrie M.L., Yuan N.Y., Kodali R., Jahan R., Emala C.W., Cook J.M. (2016). A New Pharmacological Approach for Asthma through Tissue-Specific Modulation of the GABA(A) Receptor. J. Allergy Clin. Immunol..

[14] Hagan D.W., Ferreira S.M., Santos G.J., Phelps E.A. (2022). The role of GABA in islet function. Front. Endocrinol..

[15] Ramesh S.A., Tyerman S.D., Gilliham M., Xu B. (2017). γ-Aminobutyric acid (GABA) signalling in plants. Cell. Mol. Life Sci..

[16] Breitkreuz K.E., Shelp B.J., Fischer W.N., Schwacke R., Rentsch D. (1999). Identification and characterization of GABA, proline and quaternary ammonium compound transporters from *Arabidopsis thaliana*. FEBS Lett..

[17] Ji J., Shi S.Q., Chen W., Xie T.T., Du C.J., Sun J.C., Shi Z., Gao R.F., Jiang Z.P., Xiao W.F. (2020). Effects of exogenous γ-Aminobutyric acid on the regulation of respiration and protein expression in germinating seeds of mungbean (*Vigna radiata*) under salt conditions. Electron. J. Biotechnol..

[18] Li Y., Chen Y.Z., Ma K.K., Bai M.M., Liu Y.Y., Yu X.J. (2023). Physiological changes associated with enhanced cold resistance during *Medicago ruthenica* germination and seedling growth in response to exogenous γ-aminobutyric acid. Grassl. Sci..

[19] White J.P., Prell J., Ramachandran V.K., Poole P.S. (2009). Characterization of a γ-Aminobutyric Acid Transport System of *Rhizobium leguminosarum* bv. viciae 3841. J. Bacteriol..

[20] Hijaz F., Nehela Y., Killiny N. (2018). Application of gamma-aminobutyric acid increased the level of phytohormones in *Citrus sinensis*. Planta.

[21] Mazzucotelli E., Tartari A., Cattivelli L., Forlani G. (2006). Metabolism of γ-aminobutyric acid during cold acclimation and freezing and its relationship to frost tolerance in barley and wheat. J. Exp. Bot..

[22] Li Z., Yu J., Peng Y., Huang B. (2016). Metabolic pathways regulated by γ-aminobutyric acid (GABA) contributing to heat tolerance in creeping bentgrass (*Agrostis stolonifera*). Sci. Rep..

[23] Wang Y., Xiong F., Nong S., Liao J., Xing A., Shen Q., Ma Y., Fang W., Zhu X. (2020). Effects of nitric oxide on the GABA, polyamines, and proline in tea (*Camellia sinensis*) roots under cold stress. Sci. Rep..

[24] Yang R., Guo Q., Gu Z. (2013). GABA shunt and polyamine degradation pathway on γ-aminobutyric acid accumulation in germinating fava bean (*Vicia faba* L.) under hypoxia. Food Chem..

[25] Ji H., Lu X.Y., Zong H., Zhuge B. (2018). γ-aminobutyric acid accumulation enhances the cell growth of *Candida glycerinogenes* under hyperosmotic conditions. J. Gen. Appl. Microbiol..

[26] Feng D., Gao Q., Sun X., Ning S., Qi N., Hua Z., Tang J. (2023). Effects of foliage-applied exogenous *γ*-aminobutyric acid on seedling growth of two rice varieties under salt stress. PLoS ONE.

[27] Chen X., Li N., Liu C.L., Wang H.T., Li Y.X., Xie Y.M., Ma F.W., Liang J.K., Li C.Y. (2022). Exogenous GABA improves the resistance of apple seedlings to long-term drought stress by enhancing GABA shunt and secondary cell wall biosynthesis. Tree Physiol..

[28] Wang X., Wang X., Peng C., Shi H., Yang J., He M., Zhang M., Zhou Y., Duan L. (2022). Exogenous Gamma-aminobutyric Acid Coordinates Active Oxygen and Amino Acid Homeostasis to Enhance Heat Tolerance in Wheat Seedlings. J. Plant Growth Regul..

[29] Cheng B., Li Z., Liang L., Cao Y., Zeng W., Zhang X., Ma X., Huang L., Nie G., Liu W. (2018). The γ-Aminobutyric Acid (GABA) Alleviates Salt Stress Damage during Seeds Germination of White Clover Associated with Na^+^/K^+^ Transportation, Dehydrins Accumulation, and Stress-Related Genes Expression in White Clover. Int. J. Mol. Sci..

[30] Yuan D., Wu X., Gong B., Huo R., Zhao L., Li J., Lue G., Gao H. (2023). GABA Metabolism, Transport and Their Roles and Mechanisms in the Regulation of Abiotic Stress (Hypoxia, Salt, Drought) Resistance in Plants. Metabolites.

[31] Wu X.L., Jia Q.Y., Ji S.X., Gong B.B., Li J.R., Lü G.Y., Gao H.B. (2020). Gamma-aminobutyric acid (GABA) alleviates salt damage in tomato by modulating Na^+^ uptake, the *GAD* gene, amino acid synthesis and reactive oxygen species metabolism. BMC Plant Biol..

[32] Nayyar H., Kaur R., Kaur S., Singh R. (2014). γ-Aminobutyric Acid (GABA) Imparts Partial Protection from Heat Stress Injury to Rice Seedlings by Improving Leaf Turgor and Upregulating Osmoprotectants and Antioxidants. J. Plant Growth Regul..

[33] Li D.H., Zhang D.P., Zhang Z.Z., Xing Y.M., Sun N., Wang S., Cai H. (2022). Exogenous Application of GABA Alleviates Alkali Damage in Alfalfa by Increasing the Activities of Antioxidant Enzymes. Agronomy.

[34] Zhou H., Chen H., Bao D., Shin T.Y., Zhong Y., Zhang X., Wu Y. (2022). Recent advances of γ-aminobutyric acid: Physiological and immunity function, enrichment, and metabolic pathway. Front. Nutr..

[35] Shelp B.J., Bozzo G.G., Trobacher C.P., Zarei A., Deyman K.L., Brikis C.J. (2012). Hypothesis/review: Contribution of putrescine to 4-aminobutyrate (GABA) production in response to abiotic stress. Plant Sci..

[36] Mei X., Chen Y., Zhang L., Fu X., Wei Q., Grierson D., Zhou Y., Huang Y., Dong F., Yang Z. (2016). Dual mechanisms regulating *glutamate decarboxylases* and accumulation of gamma-aminobutyric acid in tea (*Camellia sinensis*) leaves exposed to multiple stresses. Sci. Rep..

[37] Liu X., Hu X.M., Jin L.F., Shi C.Y., Liu Y.Z., Peng S.A. (2014). Identification and transcript analysis of two glutamate decarboxylase genes, *CsGAD*1 and *CsGAD*2, reveal the strong relationship between *CsGAD*1 and citrate utilization in citrus fruit. Mol. Biol. Rep..

[38] Takayama M., Koike S., Kusano M., Matsukura C., Saito K., Ariizumi T., Ezura H. (2015). Tomato Glutamate Decarboxylase Genes *SlGAD2* and *SlGAD3* Play Key Roles in Regulating γ-Aminobutyric Acid Levels in Tomato (*Solanum lycopersicum*). Plant Cell Physiol..

[39] Akcay N., Bor M., Karabudak T., Ozdemir F., Turkan I. (2012). Contribution of Gamma amino butyric acid (GABA) to salt stress responses of *Nicotiana sylvestris* CMSII mutant and wild type plants. J. Plant Physiol..

[40] Al-Quraan N.A., Sartawe F.A.-B., Qaryouti M.M. (2013). Characterization of γ-aminobutyric acid metabolism and oxidative damage in wheat (*Triticum aestivum* L.) seedlings under salt and osmotic stress. J. Plant Physiol..

[41] Hu X., Xu Z., Xu W., Li J., Zhao N., Zhou Y. (2015). Application of γ-aminobutyric acid demonstrates a protective role of polyamine and GABA metabolism in muskmelon seedlings under Ca(NO_3_)_2_ stress. Plant Physiol. Biochem..

[42] Pannerchelvan S., Rios-Solis L., Wong F.W.F., Zaidan U.H., Wasoh H., Mohamed M.S., Tan J.S., Mohamad R., Halim M. (2023). Strategies for improvement of gamma-aminobutyric acid (GABA) biosynthesis via lactic acid bacteria (LAB) fermentation. Food Funct..

[43] Mleczek M., Siwulski M., Rzymski P., Budka A., Kalac P., Jasniska A., Gasecka M., Budzynska S., Niedzielski P. (2018). Comparison of elemental composition of mushroom *Hypsizygus marmoreus* originating from commercial production and experimental cultivation. Sci. Hortic..

[44] Liu M., Meng G., Zhang J., Zhao H., Jia L. (2016). Antioxidant and Hepatoprotective Activities of Mycelia Selenium Polysaccharide by *Hypsizigus marmoreus* SK-02. Biol. Trace Elem. Res..

[45] Yan P.-S., Cao L.-X., Zhang B.-Z. (2014). Efficient Purification of Antiproliferative Polysaccharides from *Hypsizigus marmoreus* with Radial Flow Chromatography. Biotechnol. Prog..

[46] Lam S.K., Ng T.B. (2001). Hypsin, a novel thermostable ribosome-inactivating protein with antifungal and antiproliferative activities from fruiting bodies of the edible mushroom *Hypsizigus marmoreus*. Biochem. Biophys. Res. Commun..

[47] Hae J.R., Min Y.U., Ji Y.A., Chang H.J., Dam H., Tae W.K., Tae Y.H. (2011). Anti-obesity Effect of *Hypsizigus marmoreus* in High Fat-fed Mice. J. Korean Soc. Food Sci. Nutr..

[48] Bao H., You S. (2011). Molecular Characteristics of Water-Soluble Extracts from *Hypsizigus marmoreus* and Their in Vitro Growth Inhibition of Various Cancer Cell Lines and Immunomodulatory Function in Raw 264.7 Cells. Biosci. Biotechnol. Biochem..

[49] Qi Y., Zhang R., Zhang M., Wen Q., Shen J. (2021). Effects of exogenous ascorbic acid on the mycelia growth and primordia formation of *Pleurotus ostreatus*. J. Basic Microbiol..

[50] You H., Wu C., Xu Y., Hang J., Yang G., Xu J. (2023). Effects of exogenous GABA on physiological indexes of mycelial of *lentinus edodes* under different culture temperatures. North. Hortic..

[51] Hou Z.Q., Zhao L., Wang Y.T., Liao X.J. (2019). Purification and Characterization of Superoxide Dismutases from Sea Buckthorn and Chestnut Rose. J. Food Sci..

[52] Chagas R.M., Silveira J.A.G., Ribeiro R.V., Vitorello V.A., Carrer H. (2008). Photochemical damage and comparative performance of superoxide dismutase and ascorbate peroxidase in sugarcane leaves exposed to paraquat-induced oxidative stress. Pestic. Biochem. Physiol..

[53] Wu L., Zhang Y., Jiang Q., Zhang Y., Ma L., Ma S., Wang J., Ma Y., Du M., Li J. (2023). Study on CAT activity of tomato leaf cells under salt stress based on microhyperspectral imaging and transfer learning algorithm. Spectrochim. Acta Part A Mol. Biomol. Spectrosc..

[54] Livak K.J., Schmittgen T.D. (2001). Analysis of relative gene expression data using real-time quantitative PCR and the 2^−ΔΔCT^ method. Methods.

[55] Zhang Y., Xu J., Li R.F., Ge Y.R., Li Y.F., Li R.L. (2023). Plants’ Response to Abiotic Stress: Mechanisms and Strategies. Int. J. Mol. Sci..

[56] Kumari V.V., Banerjee P., Verma V.C., Sukumaran S., Chandran M.A.S., Gopinath K.A., Venkatesh G., Yadav S.K., Singh V.K., Awasthi N.K. (2022). Plant Nutrition: An Effective Way to Alleviate Abiotic Stress in Agricultural Crops. Int. J. Mol. Sci..

[57] Li C., Xu S. (2022). Edible mushroom industry in China: Current state and perspectives. Appl. Microbiol. Biotechnol..

[58] Du X.-H., Zhao Q., Yang Z.L. (2015). A review on research advances, issues, and perspectives of morels. Mycology.

[59] Money N.P. (2021). Hyphal and mycelial consciousness: The concept of the fungal mind. Fungal Biol..

[60] Zhang J., Hao H., Chen M., Wang H., Feng Z., Chen H. (2017). Hydrogen-rich water alleviates the toxicities of different stresses to mycelial growth in *Hypsizygus marmoreus*. AMB Express.

[61] Kinnersley A.M., Lin F. (2000). Receptor modifiers indicate that 4-aminobutyric acid (GABA) is a potential modulator of ion transport in plants. Plant Growth Regul..

[62] Hijaz F., Killiny N. (2019). Exogenous GABA is quickly metabolized to succinic acid and fed into the plant TCA cycle. Plant Signal. Behav..

[63] Rajput V.D., Harish, Singh R.K., Verma K.K., Sharma L., Quiroz-Figueroa F.R., Meena M., Gour V.S., Minkina T., Sushkova S. (2021). Recent Developments in Enzymatic Antioxidant Defence Mechanism in Plants with Special Reference to Abiotic Stress. Biology.

[64] Guo Z., Gong J., Luo S., Zuo Y., Shen Y. (2023). Role of Gamma-Aminobutyric Acid in Plant Defense Response. Metabolites.

[65] Lei M., Wu X., Huang C., Qiu Z., Wang L., Zhang R., Zhang J. (2019). Trehalose induced by reactive oxygen species relieved the radial growth defects of Pleurotus ostreatus under heat stress. Appl. Microbiol. Biotechnol..

[66] Tyagi S., Shumayla, Singh S.P., Upadhyay S.K., Singh S.P., Upadhyay S.K., Pandey A., Kumar S. (2019). Role of Superoxide Dismutases (SODs) in Stress Tolerance in Plants. Molecular Approaches in Plant Biology and Environmental Challenges.

[67] Pandey S., Fartyal D., Agarwal A., Shukla T., James D., Kaul T., Negi Y.K., Arora S., Reddy M.K. (2017). Abiotic Stress Tolerance in Plants: Myriad Roles of Ascorbate Peroxidase. Front. Plant Sci..

[68] Xing S.G., Jun Y.B., Hau Z.W., Liang L.Y. (2007). Higher accumulation of γ-aminobutyric acid induced by salt stress through stimulating the activity of diarnine oxidases in *Glycine max* (L.) Merr. roots. Plant Physiol. Biochem..

[69] Bao H., Chen X., Lv S., Jiang P., Feng J., Fan P., Nie L., Li Y. (2015). Virus-induced gene silencing reveals control of reactive oxygen species accumulation and salt tolerance in tomato by γ-aminobutyric acid metabolic pathway. Plant Cell Environ..

[70] Li Y., Liu B., Peng Y., Liu C., Zhang X., Zhang Z., Liang W., Ma F., Li C. (2020). Exogenous GABA alleviates alkaline stress in *Malus hupehensis* by regulating the accumulation of organic acids. Sci. Hortic..

[71] Cheng P.D., Yue Q.Y., Zhang Y.T., Zhao S., Khan A., Yang X.Y., He J.Q., Wang S.C., Shen W.Y., Qian Q. (2023). Application of γ-aminobutyric acid (GABA) improves fruit quality and rootstock drought tolerance in apple. J. Plant Physiol..

[72] Aydin S., Naghshiband Hassani R., Soleimani Aghdam M. (2021). Exogenous application of GABA retards cap browning in Agaricus bisporus and its possible mechanism. Postharvest Biol. Technol..

