# Supplementary material for: Exogenous γ-Aminobutyric Acid (GABA) Enhanced Response to Abiotic Stress in Hypsizygus marmoreus by Improving Mycelial Growth and Antioxidant Capacity
Source: Metabolites. 2024 Jan 27;14(2):94. doi: 10.3390/metabo14020094 (PMC10890280; doi:10.3390/metabo14020094)
Supplement: Supplementary file 1 [file metabolites-14-00094-s001.zip › metabolites-2789797-supplementary.pdf]

metabolites

SUPPLEMENTARY MATERIAL

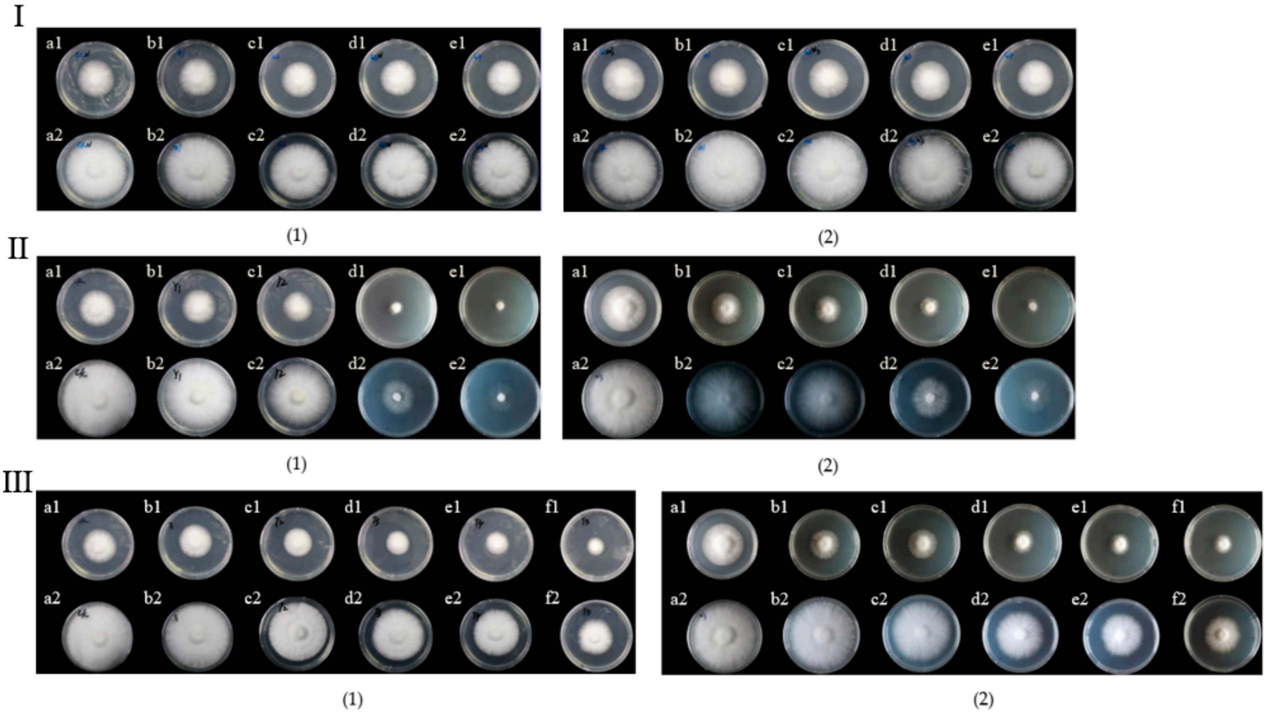

Figure S1. Effect of GABA on colony morphology of *H.marmoreus* upon addition of GABA(I), under salt stress (II) or under dehydration stress (III). (I) a1-e1 are photos of NN12 (1) and W3 (2) mycelial growth on the 7th day (medium was added with 0, 5, 10, 15, 20 mM GABA respectively), and a2-e2 are parallel photos on the 14 day; (II) a1-e1 are photos of NN12 (1) and W3 (2) mycelial growth on the 7th day (medium was added with 0, 50, 100, 150, 200, 400 mM NaCl respectively) , and a2 - e2 are parallel photos on the 14 day; (III) a1-e1 are photos of NN12 (1) and W3 (2) mycelial growth at the 7th day (medium was added with 0, 100, 200, 300, 400, 500 mM $C_6H_{14}O_6$ respectively) and a2-e2 are parallel photos on the 14 day.

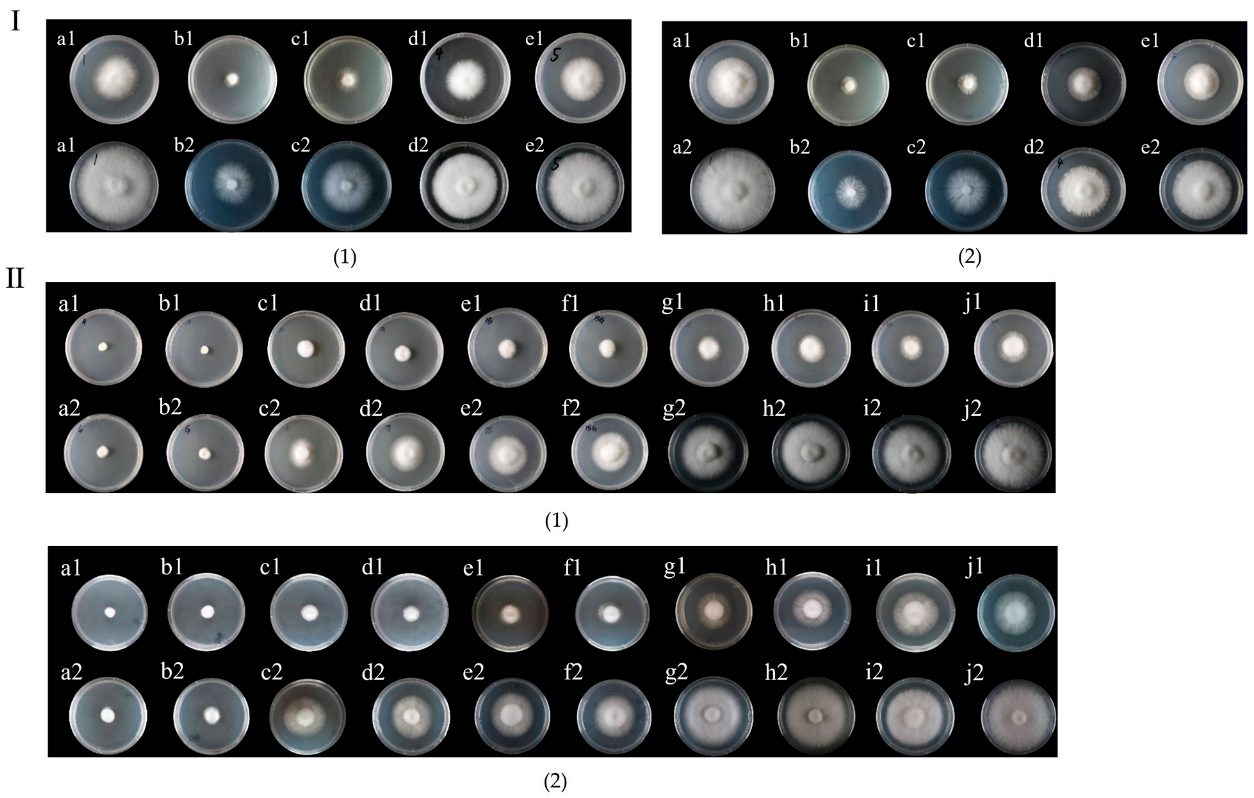

Figure S2. Effect of GABA on colony morphology under abiotic stress. (I): photos of NN12 (1) and W3 (2) mycelial growth in five groups taken on the 7th day (a1-e1) and on the 14th day (a2-e2): (a), 150 mM NaCl (b), 150 mM NaCl+ 5 mM GABA (c), 300 mM $C_6H_{14}O_6$ (d) and 300 mM $C_6H_{14}O_6$ + 5 mM GABA (e); (II): photos of NN12 (1) and W3 (2) mycelial growth taken on the 7th day (a1-j1) and on the 14th day (a2-j2): a, c, e, g, i are photos of groups at 4°C, 10°C, 15°C, 20°C, and 23°C, without GABA addition; b, d, f, h, and j are photos of groups at 4°C, 10°C, 15°C, 20°C, and 23°C added with 5 mM GABA.

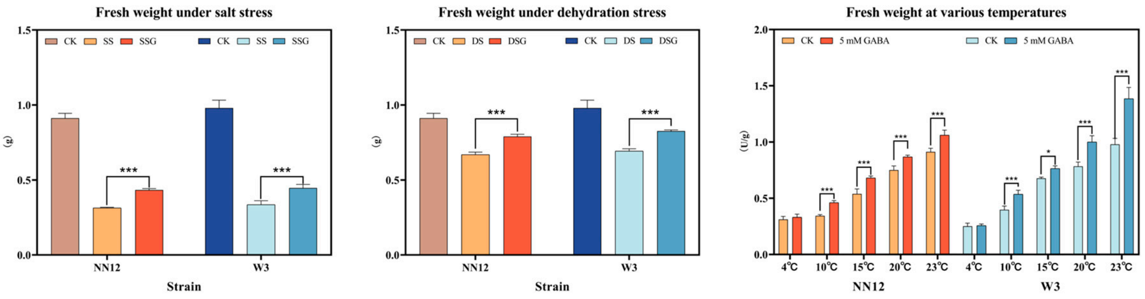

(1) (2) (3)

Figure S3. Effect of GABA on fresh weight of aerial mycelium under abiotic stress. (1) Fresh mycelium weight of NN12 and W3 under salt stress; (2) Fresh mycelium weight of NN12 and W3 under dehydration stress; (3) Fresh weight of NN12 and W3 mycelium at different temperatures. CK: control; SS: 150mM NaCl; SSG: 150mM NaCl + 5mM GABA; DS: 300Mm $C_6H_{14}O_6$; DSG: 300Mm $C_6H_{14}O_6$+5mM GABA.

Table S1. Mycelial tip diameter under salt stress and dehydration stress

Treatment group	Mycelial tip diameter(μm)
CK	6.62±0.14^{a}
150 mM NaCl	4.55±0.07^{b}
150 mM NaCl+5 mM GABA	3.72±0.14^{c}
300 mM $C_6H_{14}O_6$	5.59±0.08^{d}
300 mM $C_6H_{14}O_6$+5 mM GABA	5.27±0.15^{e}

Data are presented as means±SD(n=10); Different lowercase letters indicate significant differences in mycelial tip diameter (p<0.01)
